# Cucurbitacin I Protects H9c2 Cardiomyoblasts against H_2_O_2_-Induced Oxidative Stress via Protection of Mitochondrial Dysfunction

**DOI:** 10.1155/2018/3016382

**Published:** 2018-02-25

**Authors:** Dong Kwon Yang, Shang-Jin Kim

**Affiliations:** Department of Veterinary Pharmacology and Toxicology, College of Veterinary Medicine, Biosafety Research Institute and Korea Zoonosis Research Institute, Chonbuk National University, Jeonju, Republic of Korea

## Abstract

Cucurbitacin I, a triterpenoid natural compound, exhibits various pharmacological properties, including anticancer, anti-inflammatory, and hepatoprotective properties. However, antioxidant effects of cucurbitacin I in cardiac cells are currently unknown. In the present study, we assessed the preventive effects of cucurbitacin I against the oxidative stress in H9c2 cardiomyoblasts. To evaluate antioxidant effects of cucurbitacin I in H9c2 cardiomyoblasts, H_2_O_2_-treated H9c2 cells were pretreated with various concentrations of the cucurbitacin I. Cell viability, reactive oxygen species (ROS) production, and apoptosis were determined to elucidate the protective effects of cucurbitacin I against H_2_O_2_-induced oxidative stress in H9c2 cells. In addition, we assessed the mitochondrial functions and protein expression levels of mitogen-activated protein kinases (MAPKs). Cucurbitacin I prevented the cells against cell death and ROS production and elevated the antioxidant protein levels upon oxidative stress. Furthermore, cucurbitacin I preserved the mitochondrial functions and inhibited the apoptotic responses in H_2_O_2_-treated cells. Cucurbitacin I also suppressed the activation of MAPK proteins (extracellular signal-regulated kinase 1/2, c-Jun N-terminal kinase, and p38). Collectively, cucurbitacin I potentially protects the H9c2 cardiomyoblasts against oxidative stress and further suggests that it can be utilized as a therapeutic agent for the prevention of oxidative stress in cardiac injury.

## 1. Introduction

Ischemic heart disease (IHD), also known as coronary artery disease, is the most common type of cardiovascular disease, which occurred by reduced blood supply to the heart [1]. It is the most prevalent cause of death worldwide, especially in developed countries. Indeed, 110 million people are affected with ischemic heart disease and it resulted in 8.9 million deaths which make up 15.9% of all dead people [2, 3]. Until now, one of the effective approaches for IHD is the surgical intervention which restores the blood flow to the ischemic region. However, restoration of blood flow paradoxically causes to cardiac tissue injury known as myocardial ischemia/reperfusion (I/R) injury [4, 5]. Accumulating evidences indicate that major pathological events associated with I/R injury are oxidative stress, lipid peroxidation, intracellular calcium overload, and mitochondrial dysfunction [6]. Among them, oxidative stress which causes to accumulation of reactive oxygen species (ROS) plays a major role in the development of cardiac I/R injury [7]. Excessive ROS production leads to increased mitochondrial permeability and in turn induces apoptosis in cardiac cells, which further progresses to the chronic heart failure [8].

Recently, many natural compounds have been identified for their potential antioxidant properties by modulating the activity of antioxidant enzymes and survival signaling pathways in cardiac cells [9]. Of these, quercetin, a flavonoid found in wine, tea, and plants, has been intensively investigated for its antioxidant effect in H_2_O_2_-induced oxidative stress [10] and doxorubicin-induced cardiac injury in H9c2 cardiomyoblasts [11]. These studies demonstrated that the treatment of quercetin inhibited apoptosis, ROS production, and lipid peroxidation by modulating mitogen-activated protein kinase (MAPK) activity. Several studies also reported the beneficial actions of resveratrol, a polyphenol contained in grapes, red wine, and peanuts, in I/R-injured cardiomyocytes [12] and against the cardiotoxicity induced by various chemotherapeutic drugs, including doxorubicin and arsenic trioxide [13]. These cardioprotective roles of resveratrol are dependent upon activation of either AMPK or Sirt1 pathway [14, 15].

Cucurbitacins are triterpenoids that originally isolated from the Cucurbitaceae family plants and other plant types, such as cabbage, cucumber, melon, and watermelon [16, 17]. More than 40 members of cucurbitacin and their derivatives have been isolated, and cucurbitacin B, E, D, and I have been receiving special interests because of their relative abundance in various plants [16]. Cucurbitacins exhibit various biological and pharmacological activities, including antitumor, anti-inflammatory, hepatoprotective, and cytotoxic effects [18–20]. Particularly, most cucurbitacins, such as A, B, E, and I, affect the growth of various human cancer cell lines including breast, prostate, and brain cancer cells [21–23]. In addition, recent study also demonstrated that cucurbitacin B and I exert the preventive effects of adipocyte differentiation by modulating STAT3 signaling pathway [24].

In the present study, we first demonstrated that cucurbitacin I (Cu I) protects against H_2_O_2_-induced oxidative stress in H9c2 cardiomyoblasts and further determined that it could preserve the mitochondrial function and impaired the MAPK signaling for its underlying mechanisms.

## 2. Materials and Methods

### 2.1. Cell Culture and Oxidative Stress Induced by H_2_O_2_

H9c2 cells were purchased from the Korea Cell Line Bank (Seoul, Korea) and cultured in Dulbecco's modified Eagle's medium (GIBCO-BRL, Grand Island, NE, USA) supplemented with 10% fetal bovine serum (GIBCO-BRL) and 1% antibiotics (GIBCO-BRL) at 37°C in 5% CO_2_. Cucurbitacin I was obtained from Sigma Aldrich Co. (St. Louis, MO, USA) and dissolved in dimethyl sulfoxide (DMSO, Sigma). Cells were cultured in serum-free medium for at least 2 h, treated with 0.1, 0.5, and 1 *μ*M Cu I for 24 h, and then treated with 500 *μ*M H_2_O_2_ for 24 h to induce the oxidative stress.

### 2.2. Cell Viability Assay

Cell viability was assessed using the 3-[4, 5-dimethylthiazol-2-yl]-2, 5-diphenyltetrazolium bromide (MTT; Sigma) assay. Briefly, H9c2 cells were seeded at a density of 2000 cells per well in 96-well plates and cultured until 80% confluence. Cells were then treated with 500 *μ*M H_2_O_2_ alone or were pretreated with 0.1, 0.5, and 1 *μ*M Cu I followed by treatment of 500 *μ*M H_2_O_2_ for 24 h in triplicate. After 24 h, 0.5 mg/mL MTT was added to each well. After incubation at 37°C for 2 h, the supernatants were removed, and the formazan crystals were dissolved in 100 *μ*L DMSO. Absorbance was measured at 570 nm using a spectrophotometer (Spectra Max M5; Molecular Devices, Sunnyvale, CA, USA).

### 2.3. Measurement of ROS Production

Intracellular ROS production was detected by the fluorescence intensity of DCF-DA (2′, 7′-dichlorofluorescin-diacetate; ThermoFisher Scientific Inc., Waltham, MA, USA). Briefly, 1 × 10^5^ cells per well in 6-well plates were treated with 500 *μ*M H_2_O_2_ alone or were pretreated with 0.1, 0.5, and 1 *μ*M Cu I for 24 h followed by treatment of 500 *μ*M H_2_O_2_ for 24 h in triplicate. Thereafter, cells were treated with 1 *μ*M DCFH-DA for 30 min at 37°C. Cells were observed under a fluorescence microscope (IX-81; Olympus Corp., Shinjuku, Tokyo, Japan). The fluorescence intensity was calculated using a spectrophotometer (SpectraMax) at excitation and emission wavelengths of 488 nm and 515 nm, respectively.

### 2.4. Hoechst 33342 Staining

Apoptotic cells were evaluated by Hoechst 33342 staining. After pretreatment with different concentrations of Cu I (0.1, 0.5, and 1 *μ*M) for 24 h and then exposure to 500 *μ*M H_2_O_2_ for additional 24 h, cells were fixed in 4% paraformaldehyde for 30 min at room temperature and stained with 10 *μ*g/mL Hoechst 33342 (ThermoFisher Scientific Inc.) for 30 min at 37°C. The stained nuclei were observed under a fluorescence microscope (IX-81; Olympus Corp.).

### 2.5. Terminal Deoxynucleotidyl Transferase dUTP End Labeling (TUNEL) Staining

Apoptosis in H9c2 cells was assessed by TUNEL assay. Briefly, cells were then treated with 500 *μ*M H_2_O_2_ alone or were pretreated with 0.1, 0.5, and 1 *μ*M Cu I for 24 h followed by exposure to 500 *μ*M H_2_O_2_ for 24 h and were then fixed with 4% paraformaldehyde for 30 min at room temperature. TUNEL staining was performed using a Cell Death Detection kit (Roche Diagnostics, Manheim, Germany). The index of apoptosis was calculated using the formula ([number of TUNEL-positive cells/total number of cells] × 100%).

### 2.6. Mitochondrial Transmembrane Potential (MMP) Assessment

MMP was measured by staining with JC-1 (ThermoFisher Scientific Inc.). Briefly, 1 × 10^5^ cells per well in 6-well plates were treated with 500 *μ*M H_2_O_2_ alone or were pretreated with 0.1, 0.5, and 1 *μ*M Cu I for 24 h followed by treatment of 500 *μ*M H_2_O_2_ for 24 h and then incubated with 10 *μ*g/mL JC-1 for 20 min at 37°C. JC-1-labeled cells were observed under a fluorescence microscope (IX-81; Olympus Corp.). The fluorescence intensity of JC-1 was determined using a spectrophotometer (SpectraMax) with excitation and emission wavelengths of 550 nm and 600 nm, respectively, for red fluorescence, and 485 nm and 535 nm, respectively, for green fluorescence.

### 2.7. Western Blot Analysis

Cells were treated with 500 *μ*M H_2_O_2_ alone or were pretreated with 0.1, 0.5, and 1 *μ*M Cu I for 24 h followed by exposure to 500 *μ*M H_2_O_2_ for 24 h, harvested, and lysed in RIPA buffer (1% NP-40, 50 mM Tris-HCl, pH 7.4, 150 mM NaCl, and 10 mM NaF) supplemented with a protease inhibitor cocktail (ThermoFisher Scientific Inc.) and a phosphatase inhibitor cocktail (Roche Diagnostics). Protein samples were separated on SDS-PAGE and transferred to PVDF membranes (EMD Millipore Inc., Billerica, MA, USA). After blocking for 1 h with 5% bovine serum albumin (Sigma) in TBST (0.1% Tween-20 in Tris-buffered saline) at room temperature, the membranes were then incubated overnight at 4°C with antibodies against superoxide dismutase- (SOD-) 1 (Santa Cruz Biotechnology, Santa Cruz, CA, USA), catalase (Cell Signaling Tech., Danvers, MA, USA), glutathione peroxidase (GPx; Santa Cruz Biotechnology), total or phosphorylated extracellular signal-regulated kinase 1/2 (ERK 1/2; Cell Signaling Tech.), total or phosphorylated c-Jun N-terminal kinase (JNK; Cell Signaling Tech.), total or phosphorylated p38 (Cell Signaling Tech.), Bax (Cell Signaling Tech.), Bcl-2 (Santa Cruz Biotechnology), cleaved caspase 3 (Cell Signaling Tech.), and *β*-actin (Santa Cruz Biotechnology). The membranes were then incubated with the appropriate horseradish peroxidase-conjugated secondary antibodies (Cell Signaling Tech.) at room temperature for 1 h. Signals were detected using an Immobilon Western Chemiluminescent kit (Millipore Corp., Billerica, MA, USA) and a UVITEC Mini HD9 system (Cleaver Scientific Ltd., Warwickshire, UK). The intensity of each protein band was quantified using NIH Image J software.

### 2.8. Quantitative Real-Time Polymerase Chain Reaction (qRT-PCR)

Total RNA was isolated from the cells treated with 500 *μ*M H_2_O_2_ alone or pretreated with 0.1, 0.5, and 1 *μ*M Cu I followed by treatment of 500 *μ*M H_2_O_2_ for 24 h by using a Ribospin™ II kit (GeneAll Biotechnology Co., LTD, Seoul, Korea). To examine the expression levels of mitochondrial biogenesis genes, 1 *μ*g total RNA from the cells in each group was reverse transcribed into cDNA using ImProm II reverse transcriptase (Promega Co., Madison, WI, USA) with oligo-dT priming. qRT-PCR was performed using a Takara Thermal Cycler Dice Real-Time System (Takara Bio. Inc., Shiga, Japan) with SYBR Green (Takara) as a fluorescent dye. The primer sequences were as follows: peroxisome proliferator-activated receptor *α* (PPAR*α*), forward 5′-GGC AAT GCA CTG AAC ATC GAG-3′ and reverse 5′-GCC GAA TAG TTC GCC GAA AG-3′; peroxisome proliferator-activated receptor *γ* coactivator- (PGC-) 1*β*, forward 5′-GTG AGA TAG TCG AGT GCC AGG TG-3′ and reverse 5′-TTC TCA GGG TAG CGC CGT TC-3′; estrogen-related receptor *α* (ERR*α*), forward 5′-GCT GAA AGC TCT GGC CCT TG-3′ and reverse 5′-TGC TCC ACA GCC TCA GCA T-3′; nuclear respiratory factor- (NRF-) 1, forward 5′-CAC TCT GGC TGA AGC CAC CTT AC-3′ and reverse 5′-TCA CGG CTT TGC TGA TGG TC-3′; and 18S, forward 5′-TTC TGG CCA ACG GTC TAG ACA AC-3′ and reverse 5′-CCA GTG GTC TTG GTG TGC TGA-3′.

### 2.9. Statistical Analysis

Data were analyzed using a one-way analysis of variance (ANOVA) with the Bonferroni post hoc test using Prism 5.03 (GraphPad Software Inc., San Diego, CA, USA). All the results are expressed as mean ± SEM. *P* < 0.05 was considered statistically significant.

## 3. Results

### 3.1. Cytotoxicity of Cu I in H9c2 Cardiomyoblasts

**Figure 1:**
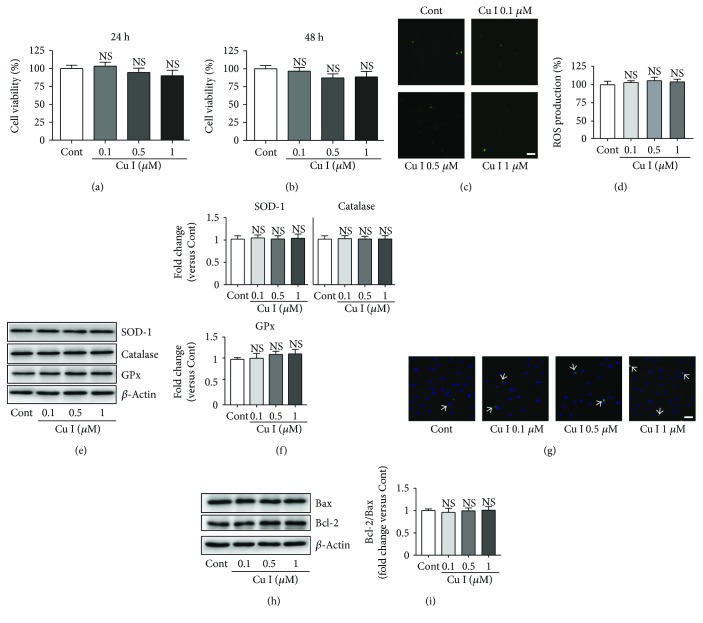
Cytotoxicity of Cu I in H9c2 cardiomyoblasts. Cell viability was measured using the MTT assay in cells treated with 0.1, 0.5, and 1 *μ*M for (a) 24 h or (b) 48 h. (c) Representative images and (d) fluorescence intensities of DCFH-DA staining assay in cells treated with 0.1, 0.5, and 1 *μ*M Cu I for 48 h. (e) Western blot analysis of SOD-1, catalase, and GPx protein expression levels in cells treated with Cu I for 48 h. (g) Representative images of TUNEL staining assay in cells pretreated with Cu I for 48 h. (h) Western blot analysis of Bax and Bcl-2 protein expression levels in Cu I-treated cells for 48 h. (f, i) Protein expression levels were quantified by scanning densitometry. *β*-Actin was used as the loading control. Western blot analysis was performed in triplicate with three independent samples. Data are expressed as the % changes ± SEM versus control cells from three independent experiments. Significance was analyzed by a one-way ANOVA followed by the Bonferroni post hoc test. Cont: control; Cu I: cucurbitacin I; NS: not significant. Scale bar: 100 *μ*m.

To determine the cytotoxic effects of Cu I in H9c2 cardiomyoblasts, we evaluated the cell viability of cells treated with 0.1, 0.5, and 1 *μ*M Cu I for 24 h and 48 h by MTT assay. Cell viability did not significantly decrease in all Cu I-treated groups compared with that in control cells (Figures 1(a) and 1(b)). ROS did not produce in cells treated with all Cu I-treated groups compared with that in control cells from the DCFH-DA staining assay (Figures 1(c) and 1(d)). Additionally, all Cu I-treated cells were shown that the protein expression of antioxidant proteins, including SOD-1, catalase, and GPx, did not change compared with those in control cells (Figures 1(e) and 1(f)). Finally, apoptosis did not induce in cells pretreated with all Cu I-treated cells compared with that in control cells from the TUNEL staining assay (Figure 1(g)) and Western blot analysis of apoptosis regulators, such as Bas and Bcl-2 (Figures 1(h) and 1(i)). Therefore, these results indicate that Cu I treatment has no cytotoxic effects on the H9c2 cardiomyoblasts.

### 3.2. Cu I Rescues H9c2 Cardiomyoblasts from H_2_O_2_-Induced Oxidative Stress

**Figure 2:**
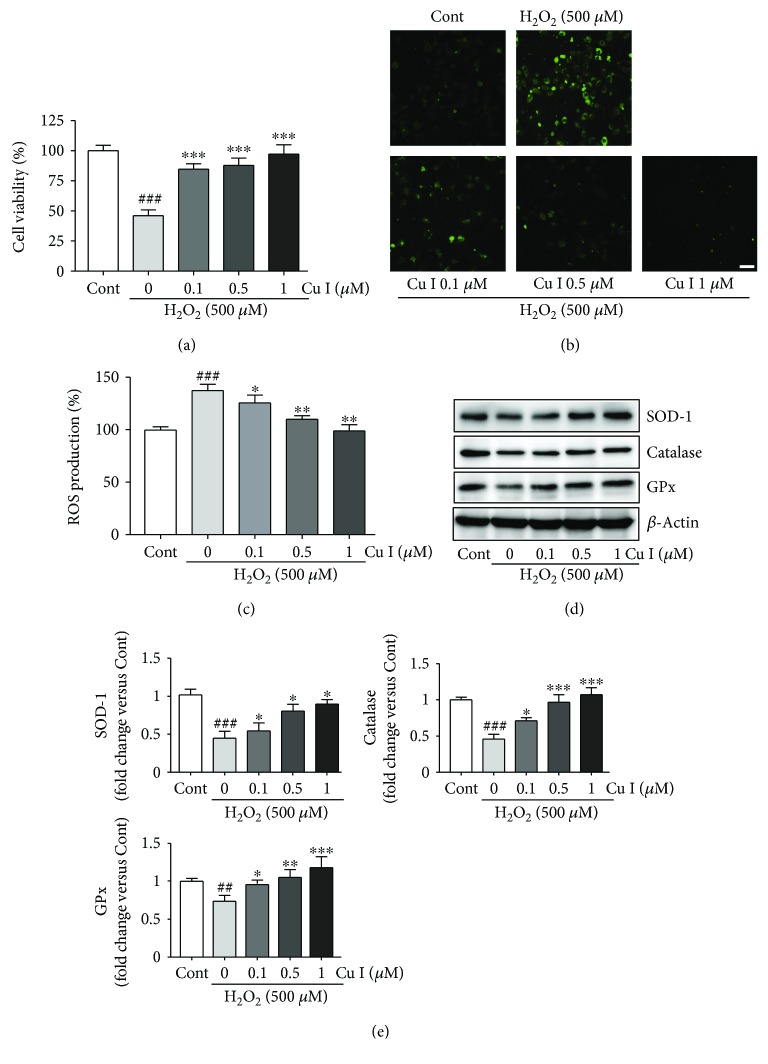
Cu I prevents the mortality and accumulation of ROS production in H_2_O_2_-treated H9c2 cardiomyoblasts. (a) Cell viability was measured using the MTT assay in cells pretreated with 0.1, 0.5, and 1 *μ*M Cu I for 24 h followed by exposure to 500 *μ*M H_2_O_2_ for additional 24 h. ROS production was assessed by DCFH-DA staining. (b) Representative images and (c) fluorescence intensities in cells pretreated with 0.1, 0.5, and 1 *μ*M Cu I for 24 h followed by exposure to 500 *μ*M H_2_O_2_ for additional 24 h. (d) Western blot analysis of SOD-1, catalase, and GPx protein expression levels. (e) Protein expression levels were quantified by scanning densitometry. *β*-Actin was used as the loading control. Western blot analysis was performed in triplicate with three independent samples. Data are expressed as fold changes ± SEM versus control cells. Significance was analyzed by a one-way ANOVA followed by the Bonferroni post hoc test. ^##^*P* < 0.01 and ^###^*P* < 0.001 versus control cells. ^∗^*P* < 0.05, ^∗∗^*P* < 0.01, and ^∗∗∗^*P* < 0.001 versus H_2_O_2_ alone-treated cells. Cont: control; Cu I: cucurbitacin I. Scale bar: 100 *μ*m.

In H_2_O_2_-treated cells, the viability was significantly decreased compared with that of control cells (46.1% decreased versus control cells). However, pretreatment with 0.1, 0.5, and 1 *μ*M Cu I for 24 h significantly increased the viability of H_2_O_2_-treated cells in a dose-dependent manner (83.6%, 90.4%, and 110.9% increases in cells pretreated with 0.1, 0.5, and 1 *μ*M Cu I versus H_2_O_2_-treated cells, resp.) (Figure 2(a)). These results indicate that Cu I prevents the mortality of H9c2 cardiomyoblasts induced by oxidative stress by H_2_O_2_ without cytotoxic effects.

### 3.3. Cu I Prevents the Accumulation of ROS Production in H_2_O_2_-Treated H9c2 Cardiomyoblasts

To determine whether Cu I can inhibit the accumulation of ROS production in H_2_O_2_-treated cells, cells were pretreated with 0.1, 0.5, and 1 *μ*M Cu I for 24 h followed by exposure to 500 *μ*M H_2_O_2_ for additional 24 h. From the staining and fluorescence assay of DCFH-DA as a ROS sensor dye, H_2_O_2_ treatment significantly increased intracellular ROS levels compared with that in control cells (158.2% increase versus control cells). Otherwise, pretreatment with Cu I dramatically decreased the ROS levels in a dose-dependent manner compared with those in H_2_O_2_ alone-treated cells (13.1%, 24.3%, and 21.4% decreases in cells pretreated with 0.1, 0.5, and 1 *μ*M Cu I versus H_2_O_2_ alone-treated cells, resp.) (Figures 2(b) and 2(c)).

Since antioxidant proteins, including SOD, catalase, and GPx, have preventive functions against oxidative stress, we further examined the expression levels of these proteins in cells pretreated with 0.1, 0.5, and 1 *μ*M Cu I for 24 h followed by exposure to 500 *μ*M H_2_O_2_ for additional 24 h. H_2_O_2_ treatment significantly decreased the expression levels of these proteins compared with those in control cells (0.43-fold, 0.45-fold, and 0.69-fold decreases in SOD1, catalase, and GPx expression versus control cells, resp.) (Figures 2(d) and 2(e)). As expected, pretreatment with Cu I significantly increased the expression of these proteins in a dose-dependent manner compared with those in H_2_O_2_-alone-treated cells (Figures 2(d) and 2(e)). Therefore, these results demonstrate that Cu I can effectively prevent accumulation of ROS production and restore the antioxidant protein levels in oxidative stress exposed-H9c2 cells.

### 3.4. Cu I Suppresses H_2_O_2_-Induced Apoptosis in H9c2 Cardiomyoblasts

**Figure 3:**
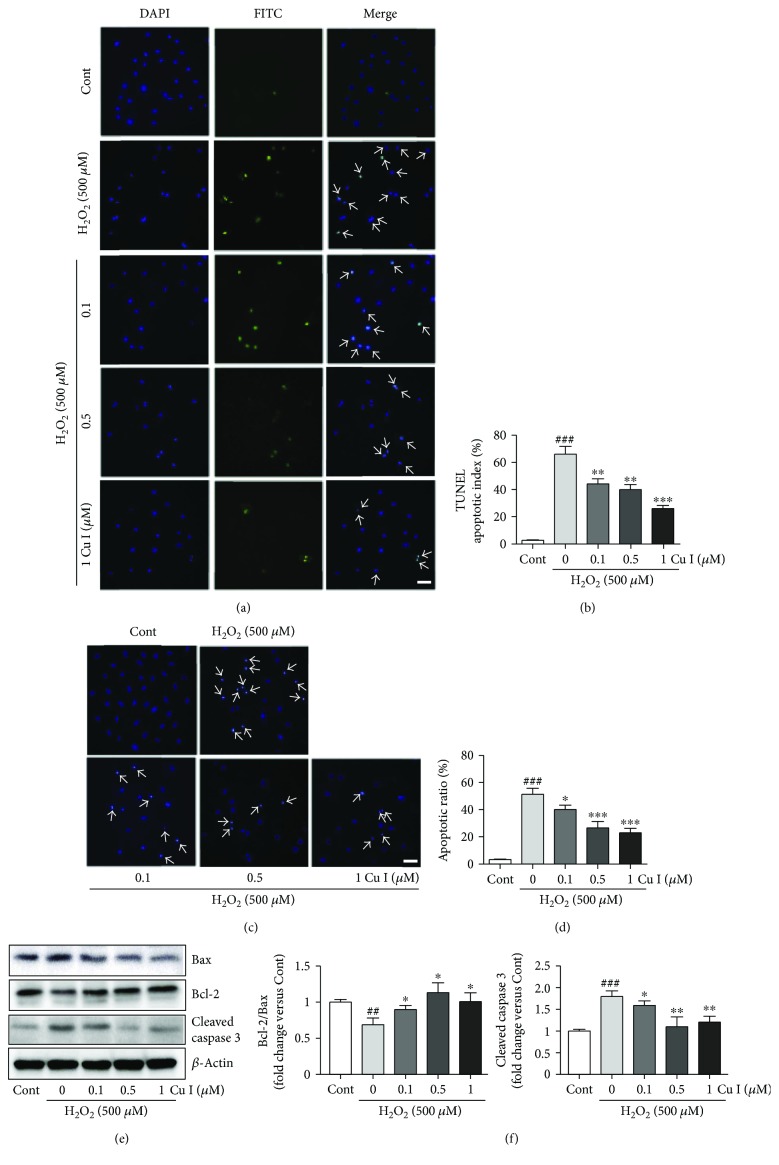
Cu I suppresses H_2_O_2_-induced apoptosis in H9c2 cardiomyoblasts. Apoptosis was determined by TUNEL, Hoechst staining, and Western blot analysis of apoptosis-related proteins. Representative images of cells pretreated with 0.1, 0.5, and 1 *μ*M Cu I for 24 h followed by exposure to 500 *μ*M H_2_O_2_ for additional 24 h in the (a) TUNEL and (c) Hoechst assays. The apoptotic index was calculated by determining the percentage of (b) TUNEL-positive or (d) Hoechst-positive cells. (e) Western blot analysis of Bax, Bcl-2, and cleaved caspase 3 protein expression levels in Cu I-pretreated/H_2_O_2_-treated cells. (f) The protein expression levels were quantified by scanning densitometry. *β*-Actin was used as the loading control. Western blot analysis was performed in triplicate with three independent samples. Data are expressed as fold changes ± SEM versus control cells. Significance was analyzed by a one-way ANOVA followed by the Bonferroni post hoc test. ^##^*P* < 0.01 and ^###^*P* < 0.001 versus control cells. ^∗^*P* < 0.05, ^∗∗^*P* < 0.01, and ^∗∗∗^*P* < 0.001 versus H_2_O_2_ alone-treated cells. Arrows indicate apoptotic cells. Cont: control; Cu I: cucurbitacin I. Scale bar, 100 *μ*m.

To assess the preventive effects of Cu I on H_2_O_2_-induced apoptosis in H9c2 cells, TUNEL and Hoechst 33342 staining were performed using the cells pretreated with 0.1, 0.5, and 1 *μ*M Cu I for 24 h followed by exposure to 500 *μ*M H_2_O_2_ for additional 24 h. TUNEL staining revealed that the percentage of TUNEL-positive cells in H_2_O_2_ alone-treated cells was much higher than that in control cells (65% increase in TUNEL-positive cells versus control cells) (Figures 3(a) and 3(b)). However, the percentage of TUNEL-positive cells in Cu I-pretreated cells was significantly lower than that in H_2_O_2_ alone-treated cells (33.8%, 38.3%, and 60.7% decreases in cells pretreated with 0.1, 0.5, and 1 *μ*M Cu I versus H_2_O_2_ alone-treated cells, resp.). Similarly, Hoechst staining showed that the percentage of apoptotic cells in H_2_O_2_ alone-treated cells was much higher than that in control cells (53% increase versus control cells). Meanwhile, the percentage of apoptotic cells in Cu I-pretreated cells was lower than that in H_2_O_2_ alone-treated cells (24.9%, 52.8%, and 58.4% decreases in cells pretreated with 0.1, 0.5, and 1 *μ*M Cu I versus H_2_O_2_ alone-treated cells, resp.) (Figures 3(c) and 3(d)). In addition, the protein expression levels of apoptosis regulators, such as Bax, Bcl-2, and cleaved caspases 3 were determined to further confirm the antiapoptotic effects of Cu I. The protein levels of Bax and cleaved caspase 3, as proapoptotic proteins, were significantly reduced in H_2_O_2_ alone-treated cells, but these increases were dramatically attenuated by pretreatment with Cu I. Otherwise, reduced levels of Bcl-2 proteins, as an antiapoptotic proteins, in H_2_O_2_ alone-treated cells were significantly preserved by pretreatment with Cu I (53.6%, 85.4%, and 69.8% increases in Bcl-2/Bax and 11.7%, 34.7%, 28.6% decreases in cleaved caspase in cells pretreated with 0.1, 0.5, and 1 *μ*M Cu I versus H_2_O_2_ alone-treated cells, resp.). Collectively, these results indicate that Cu I can prevent the apoptosis of H9c2 cardiomyoblasts induced by H_2_O_2_ (Figures 3(e) and 3(f)).

### 3.5. Cu I Prevents Mitochondrial Dysfunction upon H_2_O_2_-Induced Oxidative Stress in H9c2 Cardiomyoblasts

**Figure 4:**
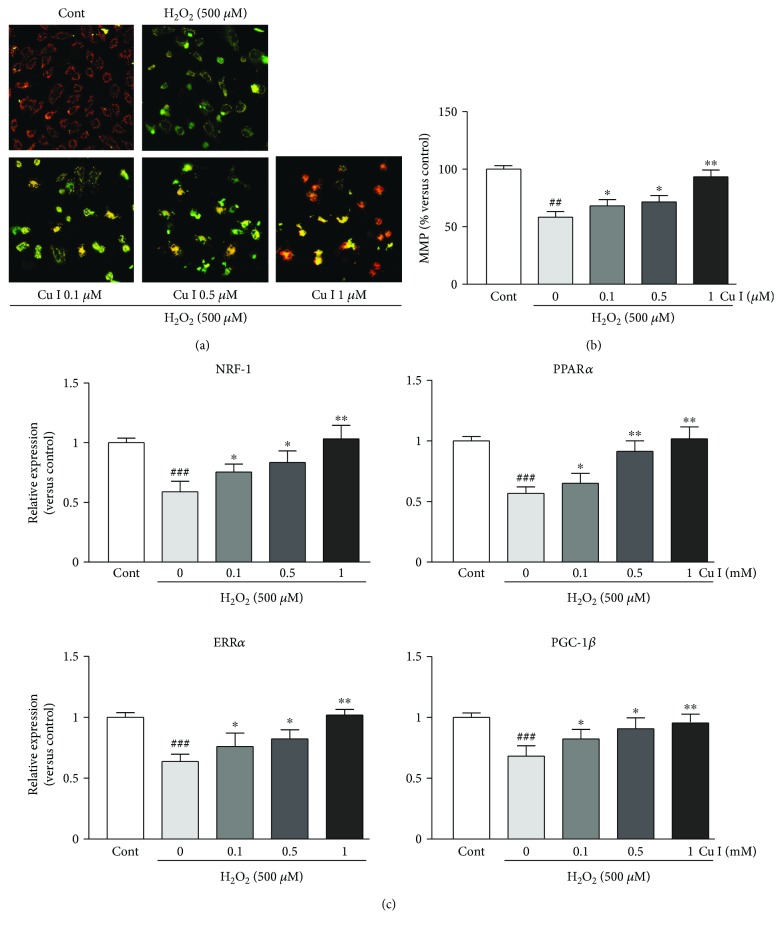
Cu I prevents mitochondrial dysfunction upon H_2_O_2_-induced oxidative stress in H9c2 cardiomyoblasts. MMP was determined by JC-1 staining. (a) Representative images and (b) fluorescence intensities in cells pretreated with 0.1, 0.5, and 1 *μ*M Cu I for 24 h followed by exposure to 500 *μ*M H_2_O_2_ for additional 24 h. (c) Quantitative RT-PCR analysis for mitochondrial biogenesis-related gene (NRF-1, PPAR*α*, ERR*α*, and PGC-1*β*) mRNA expression in Cu I-pretreated/H_2_O_2_-treated cells. The qRT-PCR analysis was performed in triplicate with three independent samples. Data are expressed as fold changes ± SEM versus control cells. Significance was analyzed by a one-way ANOVA followed by the Bonferroni post hoc test. ^##^*P* < 0.01 and ^###^*P* < 0.001 versus control cells. ^∗^*P* < 0.05 and ^∗∗^*P* < 0.01 versus H_2_O_2_ alone-treated cells. Cont: control; Cu I: cucurbitacin I. Scale bar, 100 *μ*m.

To determine the preventive effects of Cu I on H_2_O_2_-induced mitochondrial dysfunction in H9c2 cells, mitochondrial integrity was analyzed using the cells pretreated with 0.1, 0.5, and 1 *μ*M Cu I for 24 h followed by exposure to 500 *μ*M H_2_O_2_ for additional 24 h. The MMP analysis by JC-1 (MMP-sensing dye) staining revealed that MMP was dramatically reduced in H_2_O_2_ alone-treated cells compared with that in control cells (55.5% decrease versus control cells). Conversely, MMPs were significantly increased in cells pretreated with Cu I compared with those in H_2_O_2_ alone-treated cells in a dose-dependent manner (17.5%, 23.6%, and 64.7% increases in cells pretreated with 0.1, 0.5, and 1 *μ*M Cu I versus H_2_O_2_ alone-treated cells, resp.) (Figures 4(a) and 4(b)). Similarly, the mRNA expression levels of mitochondrial biogenesis-related genes, including NRF-1, PPAR*α*, ERR*α*, and PGC-1*β*, were significantly preserved by pretreatment with Cu I, while the decreased levels of these genes were shown in H_2_O_2_ alone-treated cells (Figure 4(c)).

### 3.6. Cu I Blocks the Activation of MAPK Signaling Pathway in H_2_O_2_-Treated H9c2 Cardiomyoblasts

**Figure 5:**
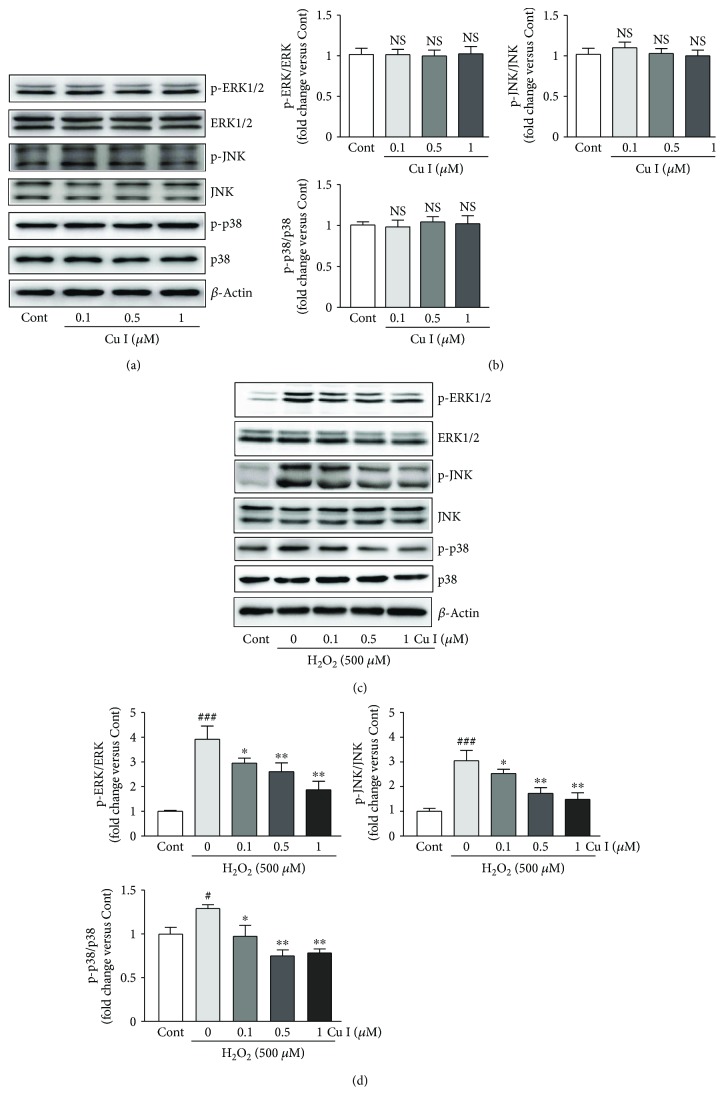
Cu I blocks the activation of MAPK signaling pathway in H_2_O_2_-treated H9c2 cardiomyoblasts. Western blot analysis of the protein expression levels of the total and phosphorylated forms of ERK1/2, JNK, and p38 in H9c2 cardiomyocytes (a) treated with Cu I for 48 h or (c) pretreated with 0.1, 0.5, and 1 *μ*M Cu I for 24 h followed by exposure to 500 *μ*M H_2_O_2_ for additional 24 h. (b, d) The protein expression levels were quantified by scanning densitometry. *β*-Actin was used as the loading control. Western blot analysis was performed in triplicate with three independent samples. Data are expressed as fold changes ± SEM versus control cells. Significance was analyzed using a one-way ANOVA followed by the Bonferroni post hoc test. ^#^*P* < 0.05 and ^###^*P* < 0.001 versus control cells. ^∗^*P* < 0.05, ^∗∗^*P* < 0.01, and ^∗∗∗^*P* < 0.001 versus H_2_O_2_ alone-treated cells. Cont: control; Cu I: cucurbitacin I.

To determine the underlying mechanisms of the protective effects of Cu I against H_2_O_2_-induced oxidative stress in H9c2 cells, the expression levels of three MAPK proteins, including ERK1/2, JNK, and p38, were analyzed by Western blotting. The results showed that treatment with 0.1, 0.5, and 1 *μ*M Cu I for 48 h did not significantly alter both phosphorylated and total forms of MAPK proteins (Figures 5(a) and 5(b)). In H_2_O_2_ alone-treated cells, the phosphorylation of ERK1/2, JNK, and p38 was significantly increased compared with that in control cells (3.9-fold, 3.1-fold, and 1.3-fold increases in p-ERK1/2/ERK1/2, p-JNK/JNK, and p-p38/p38 versus control cells, resp.). Otherwise, pretreatment with Cu I attenuated phosphorylation of these proteins in a dose-dependent manner (24.6%, 33.5%, and 52.2% decreases, p-ERK1/2/ERK1/2; 15.0%, 41.0%, 49.1% decreases, p-JNK/JNK; 25.8%, 45.6%, and 41.9% decreases, p-p38/p38 in cells pretreated with 0.1, 0.5, and 1 *μ*M Cu I, resp.) (Figures 5(c) and 5(d)). Hence, these data indicate that Cu I effectively inhibits activation of MAPKs in H_2_O_2_-treated H9c2 cardiomyoblasts.

## 4. Discussion

Increasing evidence demonstrated that oxidative stress induced by excessive ROS production is involved in the pathogenesis of various heart diseases, including ischemic heart disease, myocardial infarction, and heart failure [25, 26]. Since oxidative stress is mainly caused by imbalance between oxidants and antioxidants, antioxidant systems may play a crucial role in preventing cardiac injury, especially I/R injury. Furthermore, oxidative stress causes severe damage to the heart because heart is vulnerable to oxidative stress due to lower levels of antioxidant proteins than other organs [27]. Therefore, therapeutic strategy for preventing oxidative stress in cardiac cells is either supply of exogenous antioxidants or upregulation of endogenous antioxidants. Recently, naturally occurring bioactive compounds were intensively studied to find their antioxidant properties against the cardiac injury [9].

Cu I is one of the abundant members of cucurbitaceae family and exhibits cytotoxic and anticancer properties in various types of cancer cell lines [28–30]. Considering the pharmacological effects of Cu I against cardiac diseases, previous study showed that Cu I inhibit cardiomyocyte hypertrophy through inhibition of connective growth factor (CCN2) and transforming growth factor- (TGF-) *β*/SMAD signaling pathways [31]. Nevertheless, its antioxidant property in cardiac cells is still unknown. Hence, the present study sought to evaluate the antioxidant effects of Cu I in H_2_O_2_-treated H9c2 cardiomyoblasts.

In the present study, H_2_O_2_ as a potent oxidant, which leads to reduced cell viability, antioxidant activity, and induced apoptosis [32], was used to induce the oxidative stress in H9c2 cardiomyoblasts. The present study demonstrated that pretreatment with Cu I increased the viability of H_2_O_2_-treated cells in a dose-dependent manner, while exposure to H_2_O_2_ alone decreased the viability of H9c2 cells. In addition, inhibition of ROS production and increased expression of several antioxidant proteins (SOD-1, catalase, and GPx) were shown by pretreatment of Cu I in H_2_O_2_-induced H9c2 cells. Therefore, these results suggest that Cu I effectively protects the oxidative stress in H_2_O_2_-treated H9c2 cardiomyoblasts.

Mitochondria are major target of ROS which has the detrimental effects on the mitochondrial structure and function in cardiac injury [33, 34]. Consequently, mitochondrial dysfunction triggers the apoptosis in cardiac cells under oxidative stress condition [26]. Of note, mitochondria are rich in the heart, as a high-energy demand organ, to maintain the cardiac functions [35]. Therefore, inhibiting the mitochondrial dysfunction may be an effective way to prevent the cardiac injury caused by oxidative stress. Here, we demonstrated that pretreatment with Cu I dramatically increased MMP and preserved the expression of mitochondrial biogenesis-related genes including NRF-1, PPAR*α*, ERR*α*, and PGC-1-*β* in H_2_O_2_-exposed cells. In addition, Cu I inhibited the apoptotic responses and reduced the proapoptotic proteins (Bax and cleaved caspases 3) and increased the antiapoptotic protein (Bcl-2).

MAPK family implicated various cell functions, including proliferation, differentiation, and apoptosis [36]. When oxidative stress occurs, these MAPK proteins are activated and further stimulate the apoptotic responses [37]. Therefore, this study determined the protein expression of three major MAPK proteins, such as ERK1/2, JNK, and p38 to elucidate how Cu I protect against oxidative stress in H9c2 cardiomyoblasts. As expected, pretreatment of Cu I significantly suppressed activation of these MAPK proteins, while these were dramatically activated in H_2_O_2_-treated cells.

## 5. Conclusion

The present study demonstrated that Cu I effectively protects against oxidative stress responses, including cell viability, ROS production, mitochondrial dysfunction, and apoptosis in H_2_O_2_-treated H9c2 cardiomyoblasts. Cu I also blocks the activation of MAPK proteins, including ERK1/2, JNK, and p38. Therefore, we suggest that Cu I is a potent antioxidant drug to protect against oxidative stress in the heart.
